# Burying beetles as a model organism to study sex differences in parental care

**DOI:** 10.1007/s00040-024-01010-0

**Published:** 2024-12-01

**Authors:** T. Ratz

**Affiliations:** Department of Evolutionary Biology and Environmental Studies, University of Zurich, Winterthurerstrasse 190, 8057 Zurich, Switzerland

**Keywords:** Biparental care, Cooperation, *Nicrophorus*, Sexual conflict, Sexual dimorphism

## Abstract

In species where both sexes care for offspring, one parent—generally the female—typically provides more care than the other. While current theory offers broad predictions on the evolution of sex differences in parental care, it remains unclear whether ecological factors, such as short-term environmental variation and the intrinsic state of parents, also influence the size of existing asymmetries between parents. Here, I highlight how recent work on burying beetles (*Nicrophorus* sp.), a now well-established taxon to study biparental care, has contributed to research on sex differences in parenting. Although female burying beetles provide more care than males, the extent of this asymmetry is context-dependent as each sex constantly readjusts care depending on the surrounding environment and own state. Nevertheless, despite variation in the magnitude of the sex differences, there are still clear patterns of care specific to each species, highlighting the importance of the evolutionary history. Finally, the presence of sex differences presumably has consequences for the efficiency of parental care and can affect offspring performance. To gain a more comprehensive understanding of the life-history and environmental conditions driving sex differences in parenting, we need more natural history research on the less commonly studied *Nicrophorus* species and more work examining behavioural responses to rapid environmental changes in all taxa. Addressing these gaps will contribute to our understanding of how sexual conflict over care is resolved and how biparental cooperation persists despite asymmetries between caring parents.

## Introduction

Parental care is a fundamental component of social interactions within families (Clutton-Brock 1991; Smiseth et al. 2012). Understanding the origins and diversity of parental care behaviours provides key insights into the evolution of sociality (Wong et al. 2013; Kramer and Meunier 2019; Socias-Martínez and Kappeler 2019). Parental care is also a prerequisite for the formation of eusocial systems (Emlen 1994, 1995; Peer and Taborsky 2006; Drobniak et al. 2015). Studying the balance of cooperation and conflict among caregivers, and the factors influencing this balance, is essential for understanding the evolution of insect societies. Crucially, parental care often differs between the sexes (Kokko and Jennions 2012; West and Capellini 2016), with sex-specific variation in the amount and duration of care documented across a wide range of organisms, including both eusocial and non-eusocial insects. In eusocial species, such as hymenopterans, care is typically provided exclusively by females (Ross et al. 2013), while in termites where both sexes provide care, females generally contribute more than males (Brossette et al. 2019). In non-eusocial species, female-only care is more common than male-only care, as observed in earwigs (Kölliker and Vancassel 2007; Suzuki 2010; Meunier 2024), cockroaches (Nalepa and Bell 1997), shield bugs (Tallamy and Denno 1981; Kaitala and Mappes 1997; Tsai et al. 2015), and leaf beetles (Windsor and Choe 1994). In species with biparental care, females typically provide more care than males, as in dung beetles (Sowigi 1996; Moczek 1999) and burying beetles (Smiseth and Moore 2004; Trumbo 2007). These differences likely reflect adaptations to different care optima for each sex or evolutionary constraints that have shaped male and female care differently. Thus, examining sex differences in parenting behaviour presents an excellent opportunity to investigate the evolutionary origins and maintenance of parental care and family living.

Sex differences in parental care likely arise from differences in optimal care strategies between males and females, as is typical for sexually dimorphic traits (Fairbairn et al. 2007). These differing optima are expected to evolve when selection pressures vary between the sexes (Badyaev 2002; Cox and Calsbeek 2009). From the perspective of an individual parent, the optimal level of care depends on key factors such as parentage uncertainty and the operational sex ratio (Kokko and Jennions 2012). Consequently, any sex-specific differences in these factors should lead to differences in care provided by males and females. The sex that has greater assurance of parentage and derives relatively lower fitness benefits from increased mating effort is likely to provide more care. Furthermore, the benefits of care in terms of increased offspring fitness generally arise from the combined efforts of both parents, whereas the costs of care in terms of reduced parental survival and future reproduction are incurred individually (Trivers, 1972; Chase, 1980). Consequently, parental care is expected to be subject to strong sexual conflict, which influences the evolution of care provided by each sex (Lessells, 2012). In family living species, research aimed at understanding the causes and resolution of sexual conflict provides insights into the factors that drive cooperation between caring parents. In species that live in complex societies, research on sexual conflict can help explain the evolution of striking interspecies differences in which sex provides care—ranging from exclusively female care in Hymenoptera to biparental care in cockroaches and termites (Nalepa 1994; Park et al. 2002).

Insects are particularly useful for studying the relationship between sex differences and the maintenance of biparental care given the remarkable diversity in the types and patterns of care both across and within species. While eusocial insects have been central to research on the evolution of cooperation among family members (Kramer and Meunier 2019), non-eusocial species with facultative parental care offer complementary study systems for exploring cooperation taking place between caring parents. Importantly, many of these species provide post-hatching parental care (Costa 2006; Trumbo 2012; Gilbert and Manica 2015) and have become well-established models in parental care research. Notable examples include earwigs (Kölliker and Vancassel 2007; Honorio et al. 2024), shield bugs (Kaitala and Mappes 1997), and burying beetles (Eggert and Müller 1997; Scott 1998; Potticary et al. 2024). These species often exhibit important variation in the forms and patterns of care at both interspecific and intraspecific levels. A striking example of this variation is the extent to which each sex contributes to parental care, ranging from biparental care to female-only or male-only care, as observed in several species including brown-hooded cockroaches (Nalepa and Bell 1997; Park et al. 2002), dung beetles (Hunt and Simmons 2002; Moczek 1999), and burying beetles (Müller et al. 1998; Parker et al. 2015). Crucially, this variation in the patterns of care often depends on environmental conditions that affect the costs and benefits of caring for one sex more than the other (Alvarez and Velando 2012). Such environmental factors can either diminish or amplify sex differences in care, thereby playing a significant role in promoting or hindering parental cooperation. For instance, the intensity of sexual competition and food availability can promote or reduce parental cooperation in dung beetles and burying beetles (Moczek 1999; Hopwood et al. 2015; Ratz et al. 2021, 2022). Thus, studying intraspecific variation in care patterns provides valuable insights into the environmental conditions that may be crucial for the emergence and maintenance of biparental care.

Although current parental care theory provides general predictions about how pre-existing life histories and long-term environmental conditions shape sex differences in parental care, there has been limited research on short-term responses to environmental variation. Parental care is phenotypically plastic, with caring parents often adjusting the amount and duration of their care in response to fluctuating environmental conditions (e.g. food availability, intraspecific competition intensity, and predation risk) and intrinsic factors (e.g. energy levels, age, and prior experience). The environmental influence on parental care has been well documented (Westneat et al. 2011; Royle et al. 2014). Likewise, the adaptive value and fitness consequences of this flexibility for both parents and offspring have attracted increasing attention from empirical and theoretical research, particularly using behavioural reaction norms to study parent–offspring interactions (Smiseth et al. 2008; de Groot et al. 2023). However, there is a notable gap in research examining how flexible parental care in response to environmental variation influences sex differences in care and, consequently, the overall pattern of care. Furthermore, little is known about the adaptive value of flexible parental care and the possible fitness consequences associated with increasing or decreasing sex differences in care. As a result, our understanding of how rapid environmental changes plastically shape flexible parental care and sex roles, and whether such changes have fitness implications, remains incomplete. Addressing these gaps would clarify why parental cooperation persists or breaks down under environmental changes and help identify the conditions that favour the emergence and maintenance of biparental care.

**Fig. 1:**
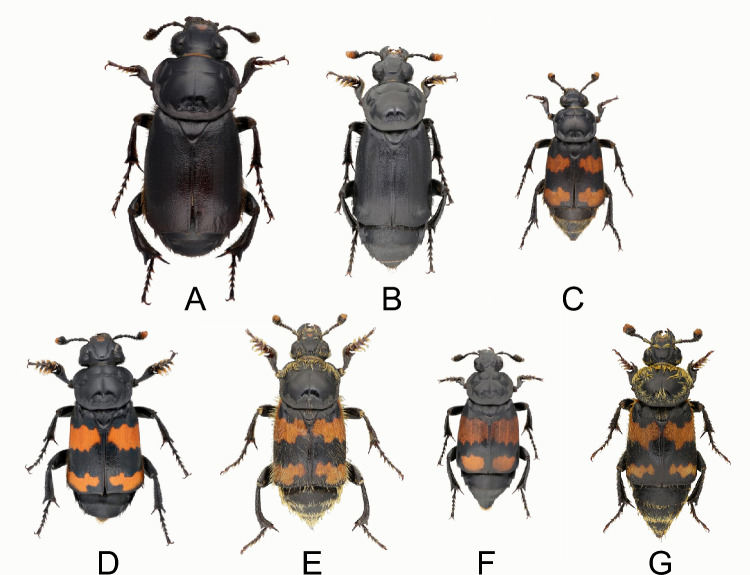
Western European species of burying beetles: *Nicrophorus germanicus* (**A**), *N. humator* (**B**), *N. interruptus* (**C**), *N. investigator* (**D**), *N. vespillo* (**E**), *N. vespilloides* (**F**), and *N. vestigator* (**G**). The large majority of *Nicrophorus* species breed on the carcass of small vertebrates and provide care to their brood, but the amount and duration of care vary considerably across species and between the sexes. Photo: Ashleigh Whiffin

Burying beetles belong to the genus *Nicrophorus*, which encompasses approximately 70 species primarily found in the northern hemisphere and southeast Asia (Sikes et al. 2002; Fig. 1). Most species have relatively short life cycles (e.g. around 6 weeks for *N. vespilloides*) and can be easily maintained under controlled laboratory conditions. Burying beetles provide extended parental care, which includes nest defence, social immunity and food provisioning (Scott 1998; Eggert and Müller 1997; Rozen et al. 2008). Care across taxa varies with some species, like *N. orbicollis*, requiring obligate post-hatching care, while others, such as *N. vespilloides* and *N. pustulatus*, have offspring that can survive without any post-hatching care (Capodeanu-Nägler et al. 2016). Although both parents can care for the larvae, females typically invest more in direct care, such as food provisioning (Smiseth and Moore 2004), as well as indirect care, including carcass maintenance (Pilakouta et al. 2018) and defence (Georgiou Shippi et al. 2018). Males tend to desert the brood earlier than females (Bartlett 1988; Scott 1998; Trumbo 1991). While biparental and female-only care is most common, instances of male-only care are also occasionally observed (Eggert and Müller 1989; Benowitz and Moore 2016). These characteristics make burying beetles an ideal system for studying the dynamics of biparental care and its fitness consequences.

In this review, I provide an overview of biparental care and sex differences in parenting among burying beetles, with a particular focus on *Nicrophorus vespilloides*, the most extensively studied species in this group. I place these case studies within the broader context of biparental cooperation and sexual conflict. I then discusse the factors driving asymmetry between the sexes, the significance of interspecific differences in the pattern of care, and what variable sex differences mean for the persistence of biparental cooperation. I give special attention to the short-term changes that influence sex asymmetry and how variable differences may impact biparental coordination and offspring performance. My goal is to illustrate how research on sex differences in parental care in burying beetles has contributed to our understanding of the evolution of biparental cooperation and identify key gaps that remain in this area of study.

## Burying beetle parental behaviour

Parental care in burying beetles is remarkable among invertebrates for its complexity and the extensive interactions it involves between parents and their larvae, as well as between male and female parents who cooperate to care for the brood. The diversity in the form and occurrence of parental care across burying beetle species makes them an excellent study system for investigating the causes and consequences of variation in care, including differences between the sexes.

Most species of burying beetles breed on the carcass of small vertebrates, typically rodents or small birds. These carcasses are valuable but scarce and ephemeral resources, leading to intense competition among burying beetles. Typically, a male and female pair will monopolise a carcass, working together to bury it and prepare it as a suitable nest for their future larvae. Carcass preparation involves removing fur or feathers, shaping the carcass into a ball, and coating it with antimicrobial and antifungal secretions to slow decay by inhibiting bacterial and fungal growth (Rozen et al. 2008; Arce et al. 2012; Trumbo et al. 2016). The processing of the carcass by parents is crucial for larval development, as the decomposing flesh serves as the primary food source for the offspring. A crucial step of carcass preparation in *N. vespilloides* also includes chewing through the skin to open a feeding incision shortly before larval hatching: this ensures that the newly hatched larvae from the eggs that are laid in the surrounding soil can access inside the carcass and rapidly start feeding on the internal organs (Eggert et al. 1998).

**Fig. 2:**
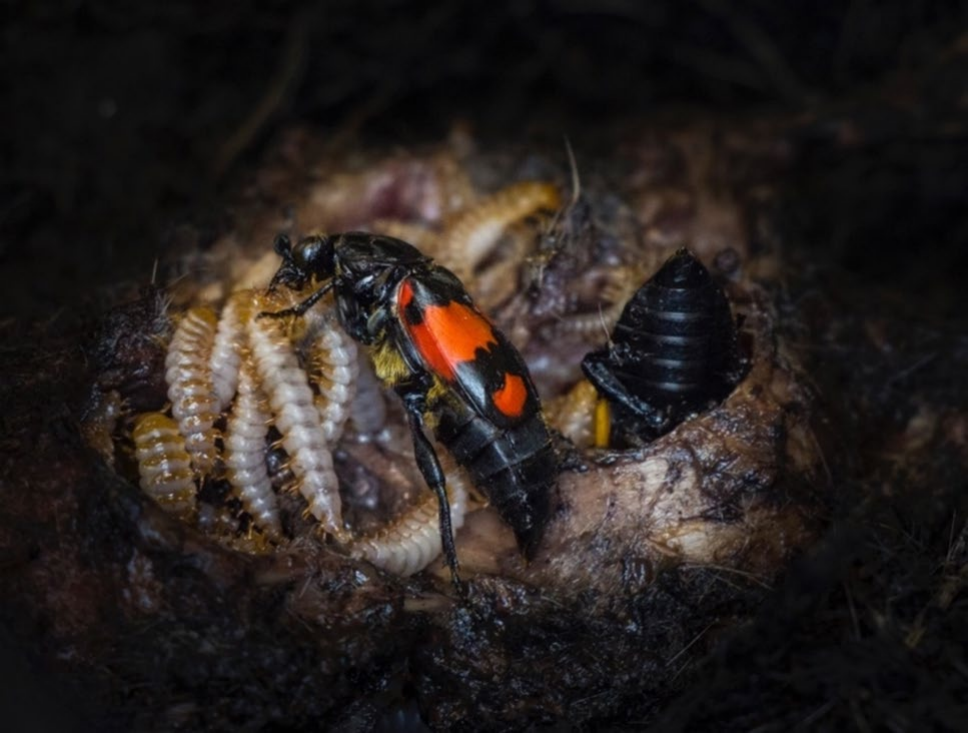
Parental provisioning in the burying beetle *Nicrophorus vespilloides*. While both parents can provide post-hatching care, females typically contribute more and usually remain until the larvae complete their development, whereas males tend to desert earlier. Post-hatching care includes food provisioning in the form of pre-digested carrion that is regurgitated to the larvae. Photo: T. Ratz

The ability of burying beetle larvae to self-feed varies across species. In *N. orbicollis* and *N. sayi*, post-hatching parental care is crucial for larval survival, as first-instar larvae cannot fully meet their nutritional needs through self-feeding and primarily rely on parental provisioning (Trumbo 1992; Capodeanu-Nägler et al. 2016). In contrast, *N. vespilloides* and *N. quadripunctatus* initially depend on food provided by the parents but can survive without post-hatching care, albeit with reduced growth and survival (Satou et al. 2001; Capodeanu-Nägler et al. 2016). Meanwhile, *N. pustulatus* larvae are entirely independent and capable of self-feeding from hatching, experiencing no detrimental effects on growth or survival in the absence of post-hatching parental care (Trumbo 1992; Capodeanu-Nägler et al. 2016, 2018). Likewise, *N. defodiens* larvae appear to develop normally without post-hatching care (Trumbo 1992); however, to my knowledge, no study under standardised conditions has yet been conducted to confirm this. Parents feed the larvae by regurgitating pre-digested flesh from the carcass (Fetherston et al. 1990; Rauter and Moore 1999; Smiseth and Moore 2002; Fig. 2). Larvae signal their need by raising their bodies towards a nearby parent and waving their legs at the parent’s mouthparts (Rauter and Moore 1999; Smiseth et al. 2003). Beyond food provisioning, parents also protect the brood by defending the carcass against competitors and intruders, such as blow flies, beetle predators, and other burying beetles that might attempt to take over the carcass and kill the brood to rear their own (Wilson et al. 1984; Scott 1990; Trumbo 1990, 1991; Sun et al. 2014).

## Sex differences in parental care

**Fig. 3:**
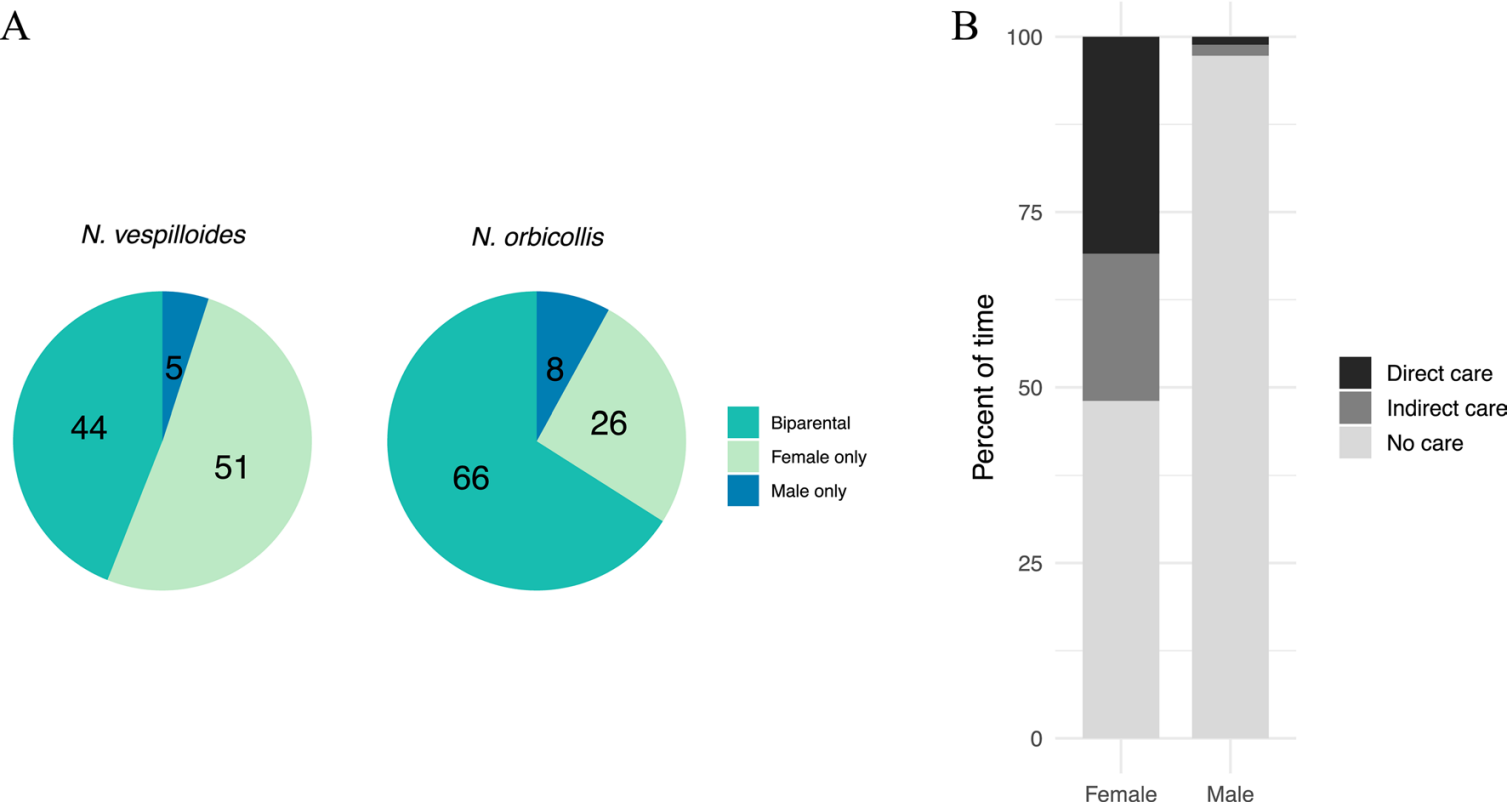
Variation in parental care in burying beetles. **A** Proportion of the different patterns of care reported in wild populations of *Nicrophorus vespilloides* and *N. orbicollis*, based on the literature (see references in the main text). Numbers inside the pie charts represent percentages. **B** Proportion of time spent providing direct care (i.e. feeding the brood) and indirect care (i.e. carcass maintenance) by each parent in a biparental care situation in *N. vespilloides*, based on data from Ratz et al. (2021)

Parental care in burying beetles varies across species in terms of pattern (uniparental vs. biparental care), and duration. In *N. vespilloides*, while the pair typically cooperates in providing pre-hatching care, males often desert earlier than females, sometimes even before the larval hatch (Eggert and Müller 1997). As a result, post-hatching biparental care is observed in approximately 44% of broods, while 51% receive care solely from the female and 5% from the male alone (Fig. 3a; Eggert 1992; Müller et al. 2007; Parker et al. 2015). In *N. orbicollis*, about 66% of broods are cared for by both parents, 26% by the female alone, and 8% by the male alone (Benowitz and Moore 2016). The variation in parental care, both among species and within species, is likely driven by life-history differences across species and individuals, including sex differences. This is supported by findings that parental care behaviours in species such as *N. orbicollis* and *N. vespilloides* are repeatable, indicating that these behaviours tend to be consistent within a species and in a given sex (Benowitz et al. 2016).

In all studied species of burying beetles, female care is generally more frequent and lasts longer than male care. This pattern is evident in species such as *N. defodiens, N. orbicollis*, *N. pustulatus*, *N. quadripunctatus*, *N. sayi*, *N. tomentosus*, and *N. vespilloides* (Bartlett 1988; Fetherston et al. 1990; Robertson 1992; Suzuki and Nagano 2009; Scott and Traniello 1990). Specifically, in *N. vespilloides*, females engage more in indirect care compared to males, including activities such as carcass preparation, maintenance, defence, and producing antimicrobial secretions and social immunity (Fig. 3b; Smiseth et al. 2005; Walling et al., 2008; Georgiou Shippi et al. 2018). In addition, females provide more direct care by spending more time regurgitating food to the larvae and consuming from the carcass, presumably to feed the larvae (Smiseth and Moore 2004; Trumbo 2007; Pilakouta et al. 2016). This consistent difference in parental care between the sexes raises important questions about the general factors that drive the evolution of sex-specific care strategies.

**Fig. 4:**
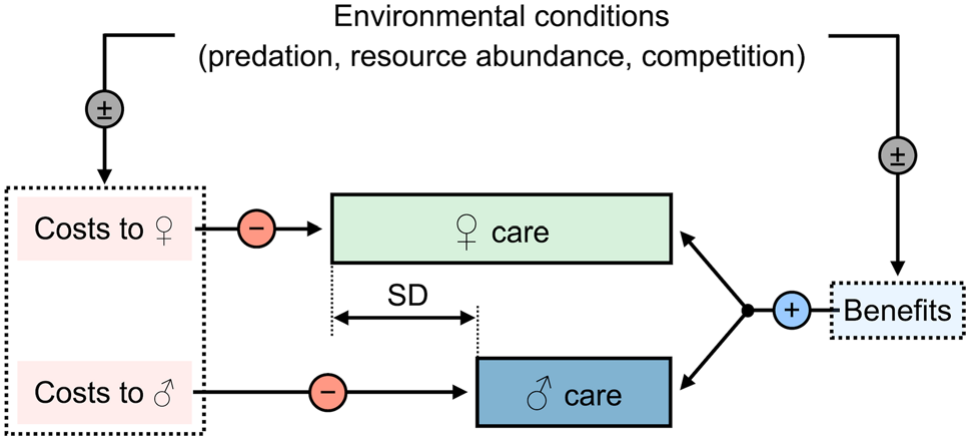
Contrasting costs and benefits of care for males and females influence the magnitude of the sex difference in parental care (SD). Each parent should adjust its care depending on the shared benefits and personal costs of caring. The benefits and costs should vary depending on environmental factors that impact the certainty of parentage, as well as the operation sex ratio that determines the prospects for remating

Sex differences in parental care likely arise from differing costs and benefits of care for males and females (Fig. 4). One of the earliest mechanisms put forward to explain this asymmetry is differential gametic investment between the sexes (Trivers 1972). In burying beetles, females invest more in gametes than males, which might suggest that they should also provide more care. However, while this mechanism may explain sex differences in care in some contexts (Long and Weissing 2023), it is logically flawed because parental care decisions should be based on future expectations rather than past investment (Kokko and Jennions 2008). Since Trivers’s seminal paper, several alternative mechanisms have been proposed to explain the differences in the costs and benefits of care between the sexes (Jennions and Kokko 2010; Klug et al. 2012; Kokko and Jennions 2012). Generally, the fitness benefits of caring for the current brood differ between the sexes due to two key factors: parentage uncertainty and the prospects for remating. In promiscuous species, males are typically less certain of their paternity compared to females, who produce the eggs. This difference in parentage certainty means that males are more likely to invest in unrelated offspring, which may lead to reduced male care (Queller 1997). In addition, males often have better prospects for increasing their fitness by obtaining additional mating partners due to higher remating success (Bateman 1948). This drives males to abandon the current brood earlier than females to seek further mating opportunities (Kokko et al. 2012; Janicke et al. 2016). This trend is particularly pronounced in female-biased populations, where females face lower chances of finding a new mate and may instead gain fitness by investing more in the current brood (McNamara et al. 2000; Kokko and Jennions 2008). It is important to note that these mechanisms are not mutually exclusive, and that the conditions for the evolution of an asymmetry in parental care may be widespread given initial differences within a population (Long and Weissing 2023).

In *N. vespilloides*, while females must secure a carcass to reproduce, males can mate away from a carcass (Müller et al. 2007). Males also tend to produce more and larger offspring after their first reproductive attempt, suggesting they may have greater residual reproductive value than females (Ward et al. 2009). Although high rates of remating increase paternity for males but not for females (Müller and Eggert 1989; House et al. 2007, 2008, 2009), parentage may play a secondary role in shaping the evolution of asymmetric parental care in burying beetles, with sexual conflict likely serving as the primary driver (Head et al. 2014). This hypothesis is especially plausible considering that females in the wild may often experience levels of parentage uncertainty similar to those of males. Conspecific brood parasitism can lead to broods where offspring are equally unrelated to both the caring female and the caring male (Müller et al. 2007). Interestingly, when males face higher reproductive competition, they tend to stay longer before deserting the brood, presumably as a tactic to protect their investment in the current brood and increase paternity through remating with the female (Hopwood et al. 2015). Burying beetle parents can also derive direct benefits from remaining at the carcass. For instance, they feed on the carcass for their own sustenance during breeding (Keppner and Steiger 2021), often gaining weight (Jenkins et al. 2000) and mitigating the effects of previous food deprivation (Trumbo and Xhihani 2015; Gray et al. 2018; Keppner et al. 2018). However, it remains unclear whether these direct benefits differ between male and female parents.

**Fig. 5:**
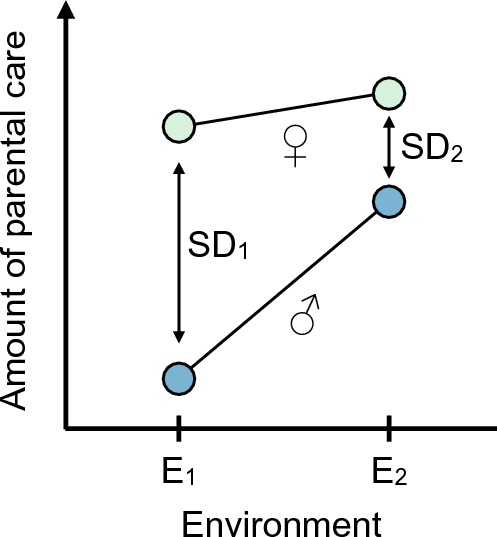
Environmental influence on the magnitude of the sex difference in parental care. Given differences in how each sex adjusts care depending on the environment, illustrated here with environments E_1_ and E_2_, the magnitude of the asymmetry can be larger (SD_1_) or smaller (SD_2_)

Beyond life-history differences, behavioural plasticity in response to short-term environmental changes also contributes to variation in parental care among burying beetles (Westrick et al. 2023). This is particularly evident in sex differences, as males and females often exhibit contrasting responses to environmental factors (Royle and Hopwood 2017; Table 1). For instance, the timing of male desertion is highly variable and depends on factors such as the size of the breeding carcass (Luzar et al. 2017; Ratz et al. 2021, 2022), the presence of the female (Smiseth et al. 2005; Royle et al. 2014), and the intensity of intraspecific competition (Hopwood et al. 2015). Males tend to show a stronger response to partner loss, increasing their amount of care more markedly than females (Rauter and Moore 2004; Smiseth et al. 2005; Suzuki and Nagano 2009; Royle et al. 2014). Furthermore, males extend their care duration when breeding on larger carcasses, a response not observed in females (Luzar et al. 2017; Ratz et al. 2021, 2022). In contrast, females are more responsive than males to variation in their nutritional state and that of their partner, reducing their level of care when paired with a well-fed mate (Lambert and Smiseth 2024). Given that females provide high levels of care regardless of the presence of a male partner, the sex differences in the plasticity of parental behaviour may reflect greater canalisation of female care compared with male care. Care may be more canalised in females because they require access to a carcass to produce offspring and thus are consistently expected to provide care (except in cases of brood parasitism). In contrast, males can achieve reproductive success by mating away from a carcass. Differences in responsiveness between the sexes suggest that the degree of the asymmetry in parental care is not fixed but varies with environmental conditions. In some environments, males and females may differ greatly in their care levels, while in others, both parents may provide more similar levels of care (Fig. 5).

**Table 1:** Summary of key topics about sex differences in parenting in burying beetles

Topic	Finding	Reference
Pattern of care	Female-only care is more common than biparental or male-only care	Eggert (1992) Müller et al. (2007) Parker et al. (2015) Benowitz and Moore (2016)
Duration of care	Males desert the brood earlier than females	Bartlett (1988) Fetherston et al. (1990) Robertson (1992) Suzuki and Nagano (2009) Scott and Traniello (1990)
Amount of indirect care (carcass maintenance, defence, social immunity)	Females provide more indirect care than males	Smiseth et al. (2005) Walling et al. (2008) Georgiou Shippi et al. (2018)
Amount of direct care (food provisioning)	Females provide more direct care than males	Smiseth and Moore (2004) Trumbo (2007) Pilakouta et al. (2016)
Flexibility in the duration of care	Males are more responsive to variation in carcass size	Luzar et al. (2017) Ratz et al. (2021)
	Males respond more to variation in the intensity of intraspecific and interspecific competition	Hopwood et al. (2015) De Gasperin et al. (2015)
Flexibility in amount of care	Males are more responsive to partner loss	Rauter and Moore (2004) Smiseth et al. (2005) Suzuki and Nagano 2009
	Females adjust indirect care depending on the presence of conspecific intruders	Ratz et al. (2022)
	Female care is more sensitive to variation in the nutritional state	Lambert and Smiseth (2024)

## Consequences of sex differences

Variation in the amount and duration of parental care can significantly impact offspring fitness. Although research on the consequences of sex differences in care is limited, evidence suggests that the sex of the caregiver influences offspring growth and survival. The quality or body condition of a parent can affect offspring performance differently depending on whether the caregiver is male or female. For instance, female body size and inbreeding status influence larval growth and survival, while male body size and inbreeding status do not (Mattey et al. 2013; Pilakouta et al. 2015; Ratz et al. 2018). This disparity may reflect greater involvement of females in care compared to males. If variation in body size and inbreeding status affects the quality of care of both sexes similarly, female care could have a greater impact on offspring performance than male care simply because females provide more care. Alternatively, variation in body size and inbreeding status may influence the quality of female care more than the quality of male care. This would be the case if smaller or inbred females provide lower quality care relative to larger or outbred females, while male care remains consistent regardless of size or inbreeding status. Distinguishing between these two explanations requires manipulating male body size and inbreeding status and monitoring offspring performance in uniparental male care situations. Given that males provide more care in the absence of a female partner (Rauter and Moore 2004; Smiseth et al. 2005; Royle et al. 2014), variation in their body size or inbreeding status would significantly impact offspring performance only if care quality is altered.

The carer’s sex not only affects offspring performance but also begging behaviour in larvae. Although offspring begging preference can reflect external influences, including the allocation of maternal hormones in the eggs (Paquet and Smiseth 2017; Paquet et al. 2020), empirical studies conducted under control conditions provide strong evidence that larvae adjust their begging behaviour to attributes of their parents. When begging preference is assessed in simultaneous choice experiment, larvae associate with and beg more towards females than male parents (Suzuki 2015; Paquet et al. 2018). Given that larvae also preferentially beg towards breeding parents when given the choice between a breeding and a non-breeding parent (Mäenpää et al. 2015), and towards larger females when given the choice between a large and a small female (Ratz et al. 2020b), it is plausible that larvae direct their begging based on expected returns. Begging is costly in terms of increased risk of mortality and energetic expenditure (Andrews and Smiseth 2013; Takata et al. 2019). Larvae should therefore maximise their returns by begging preferentially towards the parent that is more likely to provide food. Altogether, these findings suggest that interacting with females provides greater returns to offspring than interacting with males. Sex differences in parental care might thus determine the returns on begging to larvae.

The findings indicating that male presence, at least in *N. vespilloides*, does not provide additional benefits to the brood when the female is present (Jenkins et al. 2000, Smiseth et al. 2005; Ratz et al. 2018) raise the question of why males contribute any care. In fact, females alone can fulfil the entire parental role (Rauter and Moore 2004; Suzuki and Nagano 2009; Creighton et al. 2015). Although males feed from the carcass, thereby reducing the amount of food available for the female and larvae (Boncoraglio and Kilner 2012; Keppner et al. 2020), biparental care appears to confer greater overall benefits to offspring compared with uniparental care (Pilakouta et al. 2018; Trumbo 2022). For example, in *N. orbicollis*, the presence of the male during larval development increases offspring survival (Benowitz and Moore 2016; but see Trumbo and Fernandez 1995). In the wild, the male’s presence at the carcass likely plays a critical role in brood defence against predators and intruders (e.g. Eggert and Sakaluk 2000; Scott 1990; Trumbo 2006, 2007, 2022). Nevertheless, the advantage of male presence is not yet fully understood, and further research in natural settings is needed to clarify the benefits of biparental care over uniparental care in burying beetles.

Studies that directly manipulate the amount of care provided by individual parents, such as handicapping experiments, offer valuable insights into the role of paternal care and the impact of sex differences on offspring. By experimentally increasing the costs of caring—for example, by attaching a weight to the thorax–handicapping allows researchers to modify the contribution of a single parent to parental care. These experiments have been used in burying beetles to investigate how parents respond to an increase in the costs of care, and how a focal parent responds to a change in the contribution of its partner to parental care. Although both the male and the female reduce care when they are handicapped, neither sex seems to adjust care in response to their partner being handicapped when handicapping occurs after hatching (Suzuki and Nagano, 2009), and larvae do not appear to alter their begging in response to parental handicapping (Suzuki 2020). However, the timing of handicapping and the presence of a partner are important factors influencing the response to handicapping. For instance, males compensate for a reduction in female care when females are handicapped before larval hatching (Creighton et al. 2015; Suzuki 2016), and handicapped females increase their care in the absence of a partner (Ratz and Smiseth, 2018; Ratz et al. 2020a). Handicapping experiments could be further employed to amplify sex differences in care and examine how these differences might affect the brood (Wright and Cuthill 1989). For example, a fully factorial design can be used to test the effect of varying sex differences by handicapping only the male, thereby increasing sex differences, or only the female, thereby reducing sex differences, and assessing the impact of these treatments on larval growth and survival.

## Conflict and cooperation between the sexes

Biparental care involves a delicate balance of cooperation and conflict between male and female parents. The presence of sex differences in care, and the ability of parents to adjust their care plastically, provides exciting opportunities to explore the mechanisms underlying conflict resolution, cooperation, and coordination between the sexes. In burying beetles, each parent adjusts its contribution based on both the partner’s contribution and the partner’s quality or state, such as inbreeding status or body size (Mattey and Smiseth 2015; Pilakouta et al. 2015). This suggests that the outcome of coordination between parents can be influenced by experimentally manipulating the state of male and female parents (Smiseth 2019). Meanwhile, coordinating care between parents can increase the efficiency of care, reducing the costs associated with raising a brood while increasing the benefits in terms of increased offspring growth and survival (e.g. Raihani et al. 2010; Mariette et al. 2015; Bebbington and Hatchwell 2016). Although differences in parental care between the sexes might logically hinder coordination given that coordination typically requires similar workloads, it has been proposed that pre-existing differences between the sexes could instead promote task specialisation (Arnold et al. 2005). Nevertheless, the consequences of the asymmetry in care on coordination are still poorly understood. In this respect, flexible biparental care systems like those in burying beetles offer useful models for investigating how sex differences in care influence parental cooperation by influencing behavioural dynamics between caring parents.

## Concluding thoughts and perspectives

The accumulating number of studies on sex differences in burying beetles underlines the extent to which this line of research has been a stimulating topic in parental care research. Divergent care strategies between male and female parents arise from differences in key factors influencing the costs and benefits of care, such as remating opportunities and, to a lesser extent, parentage uncertainty. These sex differences reflect contrasting selection pressures acting on each sex. A key finding from studies on burying beetles is that the magnitude of sex differences is not fixed within a species but varies according to individual responses to a parent’s own state and short-term environmental variation. Males and females commonly differ in the level of their response, which implies that the size of the asymmetry between the sexes can fluctuate based on variable individual state and environmental conditions. Despite this intraspecific variation, distinct parenting behaviours across species highlight the strong influence of evolutionary history and life-history traits in shaping care patterns. Finally, while it remains unclear how sex differences might influence coordination and overall efficiency of care, the caregiver’s sex clearly impacts offspring performance. This is evidenced by work establishing that female traits, including parental care, play a more substantial role in determining offspring growth and survival than male traits.

This review highlights the need for broader research across a wider range of burying beetle species and on the short-term environmental changes that impact the costs and benefits of parental care. Family living and parental care are essential prerequisites for the development of eusocial systems (Emlen 1994, 1995; Peer and Taborsky 2006; Drobniak et al. 2015). Examining the balance between cooperation and conflict within social groups, including simple family units where two parents work together to raise offspring, is crucial for understanding how cooperation evolves in more complex social systems, such as eusocial societies. In these societies, care is sometimes provided by both sexes and sometimes only by females. Investigating the ecological and life-history conditions that affect the costs and benefits of care for each sex can provide insight into the forces shaping eusocial societies. Burying beetles, with their facultative biparental care, offer unique opportunities to examine the sex-specific costs and benefits of caring. However, most current knowledge on parental behaviour in burying beetles comes from studies on *N. vespilloides* and, to a lesser extent, *N. orbicollis*. There is a pressing need for research on less-studied *Nicrophorus* species, particularly those with poorly understood natural histories. Such research will yield valuable natural history data, enriching future comparative analyses on sex differences and sex roles.

Investigating how behavioural plasticity in response to rapid environmental changes affects sex differences in parental care is crucial to explain observed patterns of care. While current parental care theory addresses some of the long-term environmental conditions that favour the evolution of biparental care, there is a gap in understanding short-term responses to environmental variation. Consequently, the extent to which rapid plastic responses contribute to observed sex differences in care remains unclear. It also remains unclear how sex differences in parenting may have evolved through behavioural plasticity. Phenotypic plasticity can promote rapid evolutionary responses and contribute to phenotypic diversification through processes such as genetic assimilation, in which a phenotype initially induced by the environment becomes constitutively expressed (Pigliucci and Murren 2003; Lande 2009; Renn and Schumer 2013; Snell-Rood 2013). Addressing these gaps is essential to determine whether a given pattern of care primarily reflects a fixed strategy shaped by evolutionary history, or whether it results from sex-specific, flexible adjustments by parents in response to short-term environmental changes. Overall, expanding research on non-model species and rapid behavioural responses will improve our understanding of the life-history traits and environmental conditions that drive the evolution of flexible biparental care. Such effort will certainly illuminate fundamental aspects of social evolution, including the conditions necessary for the emergence of eusocial societies.

## Data Availability

There are no data associated with this paper, as it is a qualitative review of the litterature.
